# Combination of Analytical and Statistical Methods in Order to Optimize Antibacterial Activity of Clary Sage Supercritical Fluid Extracts

**DOI:** 10.3390/molecules26216449

**Published:** 2021-10-26

**Authors:** Csongor Bakó, Viktória Lilla Balázs, Gyöngyi Takács, József Péter Pallos, Szilárd Pál, Béla Kocsis, Dóra Rippelné Pethő, Györgyi Horváth

**Affiliations:** 1 Department of Pharmacognosy, Faculty of Pharmacy, University of Pécs, H-7624 Pécs, Hungary; csongor.bako@gmail.com (C.B.); balazsviktorialilla@gmail.com (V.L.B.); 2 Institute of Chemical Engineering and Process Engineering, Faculty of Engineering, University of Pannonia, H-8200 Veszprém, Hungary; strbka@almos.uni-pannon.hu (G.T.); pethod@almos.uni-pannon.hu (D.R.P.); 3 Pannonpharma Ltd., H-7720 Pécsvárad, Hungary; pallos.jp@pannonpharma.hu; 4 Department of Pharmaceutical Technology and Biopharmacy, Faculty of Pharmacy, University of Pécs, H-7624 Pécs, Hungary; pal.szilard@gytk.pte.hu; 5 Department of Medical Microbiology and Immunology, Medical School, University of Pécs, H-7624 Pécs, Hungary; kocsis.bela@pte.hu

**Keywords:** essential oils, clary sage, supercritical fluid extraction, thin layer chromatography-direct bioautography, response surface method

## Abstract

The extraction of clary sage (*Salvia sclarea* L.) using supercritical carbon dioxide (SC-CO_2_) was systematically studied by using thin layer chromatography-direct bioautography (TLC-DB) and response surface methodology (RSM). The three parameters temperature, pressure, and cosolvent ratio were optimized for the maximum antibacterial activity of clary sage extracts against *Pseudomonas aeruginosa* (*P. aeruginosa*) and methicillin-resistant *Staphylococcus aureus* (MRSA). The highest inhibition zone was 7.51 mm for *P. aeruginosa* and 7.57 mm for MRSA. According to RSM analysis, the predicted optimum extraction parameters are 18.6 MPa pressure, 40 °C temperature, and 2% ethanol (EtOH) ratio. The combination of this analytical and statistical method allows saving time, money, and instrument runtime in the optimization of essential oil composition, which is tailored to a specific task and could be useful on any kind of herbs in a wide range of use from perfume manufacturing to the food industry.

## 1. Introduction

The rapid technological development of our modern world is difficult to be followed on a human scale. In addition to the obvious benefits, the challenges ahead unfold at a similar pace. Some of these are so urgent to humanity that their solution has become the strategic direction of several leading countries. These include the protection of the environment, the fight against antibiotic resistance, and the digitalization (e.g., three dimensional statistical models to aid design).

Due to the varied composition of essential oils (EO), the bacteria find it difficult to develop resistance [1,2]. Therefore, they may serve as good alternatives to antibiotics for the pharmaceutical industry [3]. However, their use is more common in the food and perfume industries [4,5]. EO is usually a minor constituent of all the substances in a plant species, amounting up to 5% of the dried plant. The composition of an EO is highly complex, and the active components are diverse. They consist mainly of monoterpenes, sesquiterpenes, and diterpenes from isoprene-based hydrocarbons as well as oxygenated fractions of these molecules such as aldehydes, ketones, phenols, acids, alcohols, ethers, esters, etc. [6].

In the Lamiaceae family, the largest and most important herbaceous and aromatic genus is *Salvia* (sage), which contains circa 900 species that are found all over the world [7]. From the sage species, clary sage (*Salvia sclarea* L.) was chosen, which is a plant with strong aromatic scent due to the essential oil found in its flowers and leaves. It is most commonly used as a flavoring and fragrance agent in the preparation of foods and liqueurs, in the formulation of perfumes, and in the compilation of cosmetic agents [8]. The most commonly published basic ingredients of the EO include linalyl acetate, linalool, sclareol, and germacrene D. The versatility of its oil may also contribute to its antimicrobial effect, which was proven in several studies. The antibacterial effect of its active components is due to their ability to increase the permeability of the cell membrane. As a result of the leakage, macromolecules, ATP, and DNA are secreted into the extracellular space. The mode of action involves a set of mechanisms both on the cell surface and within the cytoplasm. However, the process is not completely clear, and it is evident that further research is needed to clearly understand the mechanism of action [9].

It should be mentioned that the extraction of a small amount of valuable plant material with such a diverse composition requires a well-used, easily reproducible method. Commonly used extraction methods for the isolation of the volatile fraction of medicinal plants include steam distillation, hydrodistillation, or supercritical carbon dioxide (SC-CO_2_) extraction. In practice, a high prevalence of steam distillation (SD) can be observed, which is an inexpensive and environmentally friendly method. However, hydrodistillation (HD) can cause the oxidation of certain compounds, and hydrolysis and terminal degradation has to be expected as well. These can lead to changes in the odor of the oil and the elimination of some active components [10]. Although Soxhlet extraction is very efficient, the need for using organic solvents is dangerous to the environment and to human health [11]. In contrast, supercritical fluid extraction (SFE) can be a gentle and green alternative technology to traditional extraction methods. In terms of any extraction yield, Soxhlet performs better compared to SFE, but using CO_2_ as a solvent in the latter method is cheap, harmless to the environment, and to humans. It can play a role in shaping the circular economy by reducing the generation of organic solvent waste, and CO_2_ used in extraction can either be recirculated or recovered [12]. High automation can be achieved and the optimization of a large number of parameters fits well into the methodology of experimental design [13]. The Box–Behnken design (BBD) was used in our experiments, which is a common modeling procedure for optimizing SFE methods [14].

Direct bioautography (DB) is one of the most effective methods available today to study the antimicrobial effect of multicomponent extracts. A major advantage of the method is that it combines thin layer chromatography (TLC) and microbiological assays [15]. Samples can be directly contacted with test organisms on a TLC sheet [16]. For antimicrobial activity testing, the developed chromatography sheet is immersed in a solution of bacterial suspension and incubated for an appropriate time. The bacterium grows directly on the TLC plate, except at the points where the samples exert an antibacterial effect and inhibit its growth. Then, the sheets are sprayed with tetrazolium salt, from which the viable bacteria produce a purple formazan dye [17]. This makes the zones of inhibition visible. There are few studies in the literature where specific respiratory pathogens have been studied by TLC-DB, despite the fact that studies on antibiotic resistance are becoming more common in this area. According to a World Health Organization (WHO) survey, people with methicillin-resistant *Staphylococcus aureus* (MRSA) infection are 64% more likely to die of the infection than those infected with a methicillin-sensitive bacterium [18]. The WHO recently listed carbapenem-resistant *Pseudomonas aeruginosa* among the three bacterial species for which there is a critical need in the antibiotic development field [19].

Previous research on supercritical carbon dioxide (SC-CO_2_) extraction of clary sage [20,21,22] focused on the relative proportions of the components and comparison with other extraction methods. The enrichment of the sclareol content of the extracts is also an important direction of research [23,24,25], as sclareol can be a natural substitute for ambergris for the perfume industry. The present research is a comprehensive approach to optimize clary sage SC-CO_2_ extraction conditions to achieve the greatest possible biological effect. For studies that require a large number of experiments with many variables, the time and material cost involved become critical factors. The efficiency of our use of resources can be increased by proper experimental design. Response surface methodology (RSM) is a collection of mathematical and statistical techniques that examine the relationship between one or more response variables and several explanatory variables. The individual and interactional effects of the parameters on the antibacterial activity can be modeled by the calculations [26]. The use of RSM in optimization of extraction processes is becoming more widespread [27,28,29,30,31,32].

The aim was to determine the optimal set of parameters (pressure, temperature, and cosolvent ratio) for the recovery of essential oil from clary sage using RSM. The extracts were tested on model bacteria: Gram-positive (MRSA) and Gram-negative (*P. aeruginosa*) respiratory tract pathogens. The process presented in this study can shorten and facilitate product development against pathogens. Furthermore, RSM can help understand and optimize subsequent manufacturing processes.

## 2. Results

### 2.1. Antibacterial Activity of Clary Sage Extracts

**Figure 1:**
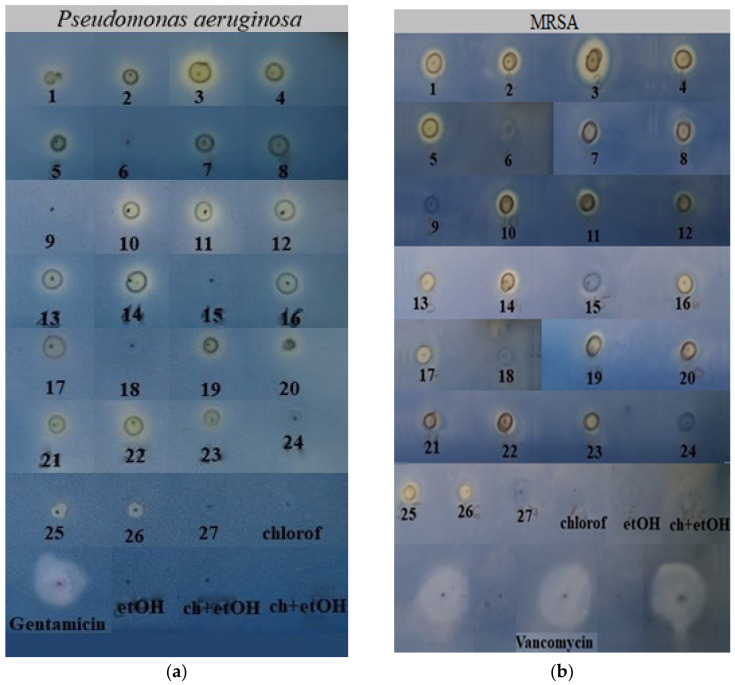
(**a**) Detection of the antibacterial activity of clary sage SC-CO_2_ extracts, positive control (gentamicin), and solvents against *P. aeruginosa* by TLC-DB. The numbering of the samples is the same as that shown in Table 1; (**b**) Detection of the antibacterial activity of clary sage SC-CO_2_ extracts, positive control (vancomycin), and solvents against MRSA by TLC-DB. The numbering of the samples is the same as that shown in Table 1.

**Figure 2:**
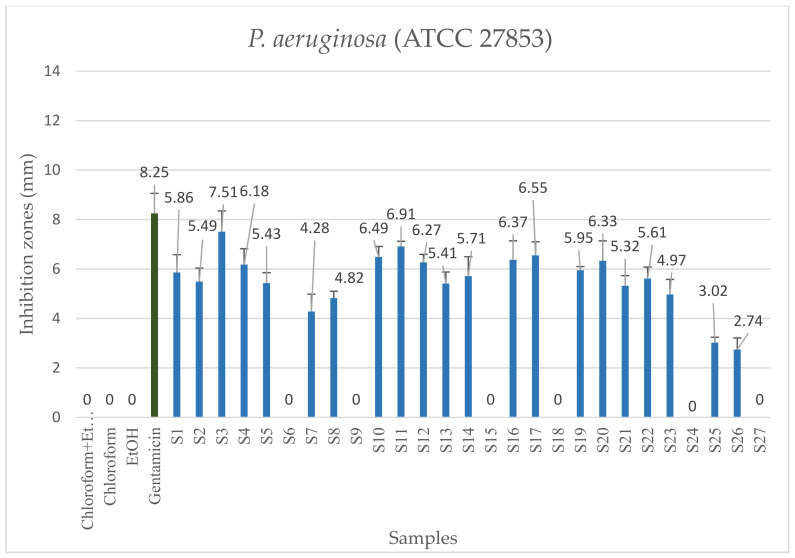
Antibacterial activity of each clary sage extracts and gentamicin as positive control on *P. aeruginosa*. The numbering of the samples is the same as that shown in Table 1.

**Figure 3:**
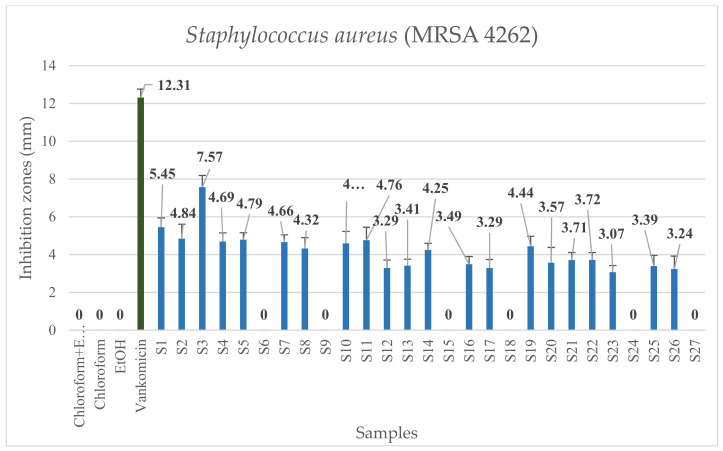
Antibacterial activity on MRSA of each clary sage extracts and vancomycin as positive control. The numbering of the samples is the same as that shown in Table 1.

Using the TLC-DB method, the antibacterial activity of the extracts without separation was tested against *P. aeruginosa* and MRSA (Figure 1a,b). Antibacterial activity was expressed in diameter of the inhibition zone (mm) (Table 1, Figure 2 and Figure 3). Chloroform and absolute ethanol as negative controls did not inhibit the growth of either bacteria. Gentamicin was used as positive control in the case of *P. aeruginosa*, which was more sensitive to this antibiotic than to any of the plant extracts. In general, the highest inhibition zones were observed for the plant samples extracted at 20 MPa. Lower temperatures also facilitated the extraction of biologically active components. Samples that did not inhibit bacterial growth were prepared at 10 MPa in each case.

In the case of MRSA, vancomycin, used as the positive control antibiotic, inhibited bacterial growth efficiently. Comparing the results obtained with MRSA to the results of *P. aeruginosa*, similar regularities were observed for the inhibition zones of the samples. However, the growth of MRSA was less inhibited by the extracts. The highest values of inhibition were observed for the third extract, the inhibition zone being 7.51 ± 0.85 mm and 7.57 ± 0.62 mm for *P. aeruginosa* and MRSA, respectively (Table 1). Low temperature (40 °C) and high cosolvent ratio (2%) were used during preparation of sample 3. Based on these observations, the antibacterial activity could be increased by the above conditions.

### 2.2. Response Surface Results

**Figure 4:**
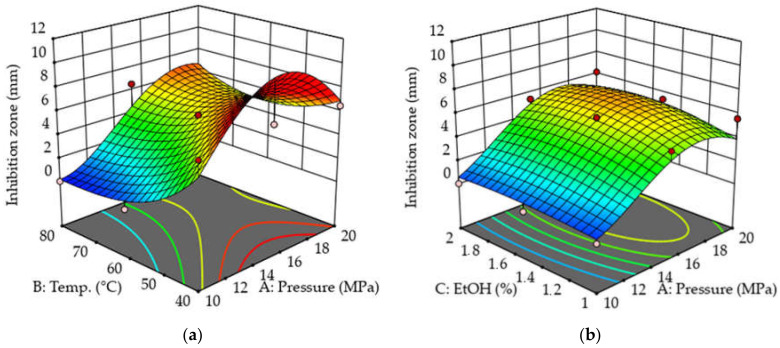
(**a**) Three-dimensional surface plot for variation of antibacterial activity on *P. aeruginosa* with changing extraction temperature and pressure. (**b**) Three-dimensional surface plot for variation of antibacterial activity on *P. aeruginosa* with modification of extraction pressure and ethanol ratio. The color scale shows the zones of inhibition from blue (0.00 mm) to red (7.51 mm).

**Figure 5:**
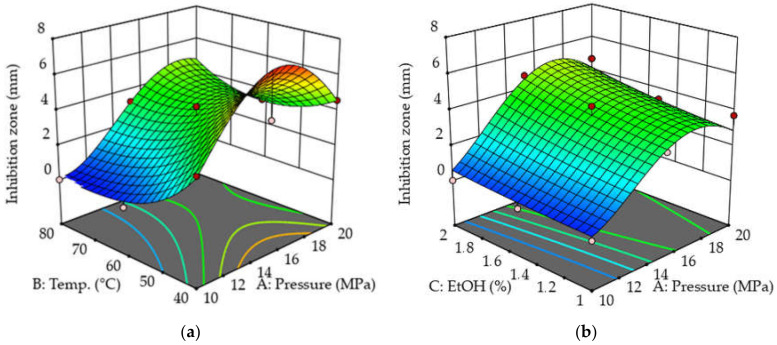
(**a**) Three-dimensional surface plot for variation of antibacterial activity on MRSA with changing extraction temperature and pressure. (**b**) Three-dimensional surface plot for variation of antibacterial activity on MRSA with extraction pressure and ethanol ratio. The color scale shows the zones of inhibition from blue (0.00 mm) to red (7.57 mm).

The response surface models of the extracts were plotted by using the Design Expert program. Figure 4 and Figure 5 show how the antibacterial activity of the extract changes against bacterial strains of *P. aeruginosa* and MRSA in addition to the constant change of certain parameters. It can be seen that in the case of *P. aeruginosa*, the diameter of the zones of inhibition was larger on average, so the extracts were more effective against this type of bacterium. The shape of the response surfaces is very similar in the two cases studied. As the figures show, the best results were obtained at low temperatures (40–45 °C) and moderately higher pressures (15–19 MPa). The least effective extracts can be prepared at 40–50 °C and 10–11 MPa. During the extraction, the plant material must be protected from excessive heat. As a function of the cosolvent ratio, the shape of the figures is less characteristic, from which it can be concluded that of the three setting parameters, the EtOH concentration has the least effect on the antibacterial activity. In the case of *P. aeruginosa*, the optimal ratio may be between 1.6 and 1.7%, and a further increase in the EtOH ratio will no longer produce a significant improvement in antibacterial activity. However, in the case of MRSA, the best results can be obtained with a cosolvent ratio of 2%. For both bacterial strains, the worst results were obtained at a ratio of 1.0–1.2% EtOH.

The results of the ANOVA tests of the two bacteria can be seen in Table 2 and Table 3. ANOVA confirmed the accuracy of the models for optimizing. The F-value is 16.40 in the case of *P. aeruginosa* and 17.90 for MRSA; this suggests that the models are significant and there may be only a 0.01% chance that a higher F-value may occur due to noise. The predicted R^2^ for *P. aeruginosa* for 0.6662 and 0.6860 MRSA reasonably corresponds to the adjusted R^2^ for *P. aeruginosa* 0.8172 and 0.8308 for MRSA, because the aberration is less than 0.2. Adeq Precision measures the signal-to-noise ratio with sufficient accuracy. A ratio greater than 4 is desirable [33]. Our ratio is 15.3486 in the case of *P. aeruginosa* and for MRSA, it is 16.3278, indicating adequate signals. The limitations mentioned above correspond to the limitations in the Design Expert software. The models can be used to navigate the design space.

**Figure 6:**
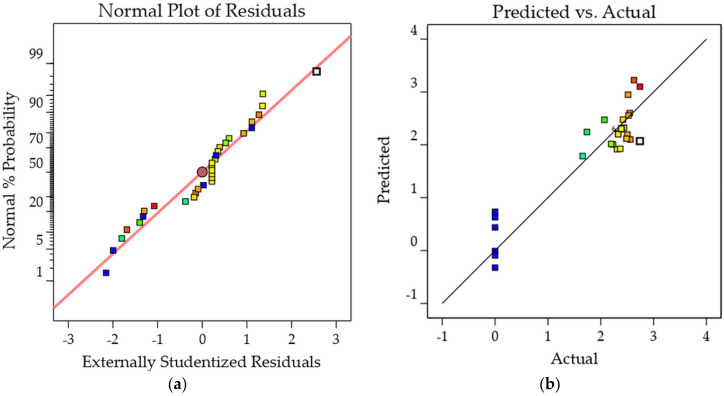
(**a**) Normal plot of residual for *P. aeruginosa* cell inhibition.; (**b**) Predicted vs. actual plot for *P. aeruginosa* inhibition. The color scale shows the value of *P. aeruginosa* in the quadratic model from blue (0.000) to red (2.740).

**Figure 7:**
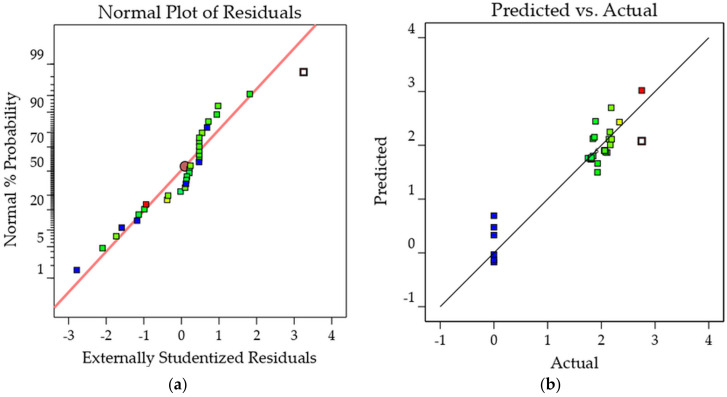
(**a**) Normal plot of residual for MRSA cell inhibition.; (**b**) Predicted vs. actual plot for MRSA inhibition. The color scale shows the value of MRSA in the quadratic model from blue (0.00) to red (2.752).

Figure 6a,b demonstrate the strong nearness between the measured values and those forecasted by the statistical calculation, as illustrated by flocking together close to the straight line. Indeed, the coefficients of determination, *P. aeruginosa* R^2^ = 0.8703 and MRSA R^2^ = 0.8799, are suitable values determining the relationship between the predicted and actual values. Figure 7a,b show the actual versus predicted values. The location of the runs near the straight line proves that the difference between the observed and the predicted values was very small [33]. The results show that the predicted and actual values are sufficiently close to each other, which validates that the models are suitable for predicting results and optimizing extraction.

*p*-values lower than 0.05 illustrate the significance of the model terms. All three setting parameters have a statistically significant effect on antibacterial activity against *P. aeruginosa*. Calculations confirmed the conclusions of the 3D figures. For the Gram-negative *P. aeruginosa*, two parameters had a statistically significant effect on antibacterial activity (*p* ˂ 0.0001). However, the ratio of EtOH had no significant effect on antibacterial activity (*p* = 0.3365). For the Gram-positive MRSA, all three setting parameters, including EtOH ratio (*p* = 0.0434), had a statistically significant effect on biological efficacy. In this case, the most significant effect was exerted by temperature and pressure on the result (*p* ˂ 0.0001). When examining the interactions between the setting parameters, only the interaction between pressure and temperature showed a statistically significant effect on the antibacterial activity (*p* ˂ 0.0002, 0.0001), while the other interactions showed no statistically significant effect at all. Based on the 3D diagrams and calculations, the choice of these two parameters (temperature and pressure) are the most critical when designing the extraction.

Optimization is an effective means of controlling processes in order to produce a product of the right quality. Optimal extraction parameters were determined to take into account the antibacterial activity for both bacteria. The treatment of multiple responses and selection of the optimal conditions were based on the desirability function D. In this research, desirability was set to calculate the best combination of setting parameters to maximize biological efficacy against *P. aeruginosa* and MRSA bacteria. Using the desirability function, the following setting parameters were determined: pressure 18.6 MPa, temperature 40 °C, and EtOH ratio 2%. Using these parameters, the diameter of the predicted inhibition zone was 7.95 mm and 7.57 mm for *P. aeruginosa* and MRSA, respectively.

## 3. Discussion

Extensive literature is available on the antimicrobial effect of EOs [1,34,35,36,37,38]. Clary sage is a widely used traditional medicinal plant that does not cause significant side effects in humans [39]. The present study demonstrates that minor changes in extraction parameters have a significant effect on the biological efficiency of the clary sage extract isolated with the SC-CO_2_ technique. At the same time, the results highlight the importance of thorough experimental design. RSM is suitable for a better understanding of extraction processes, and it helps us determine optimal process parameters.

Monitoring biological efficiency requires a method suitable for testing non-water-soluble, complex samples. There are several practical methods in the literature that are used to study the antimicrobial effect of EOs (e.g., agar dilution assay, disc diffusion assay). However, the results show great variability even during repeated measurements of a given oil [40]. The results become comparable only with standardized measurements. TLC-DB is a biological probe that combines planar chromatography with an in situ biological assay. In contrast to agar diffusion methods, EOs come into direct contact with the test bacteria, so the use of detergents and the different diffusion abilities of the components do not affect the final result.

The TLC-DB method developed by Balázs et al. was shown to be suitable for testing the anti-*Haemophilus* activity of extracts containing non-water-soluble compounds [16]. Using gathered experience, the method was optimized for the MRSA and *P. aeruginosa* strains, paying special attention to parameters previously found to be critical (e.g., incubation time, the composition of agar for growing the bacterium, etc.) that may significantly affect the efficiency of the TLC-DB method [41].

To the best of our knowledge, the extraction procedure of clary sage was optimized for the first time by our group using RSM. The antibacterial activity was first chosen as a primary target to be increased. Previous studies have mainly compared the chemical composition of the extracts depending on whether the samples were prepared by hydrodistillation or SC-CO_2_ extraction. However, the results are contradictory in many cases. Ronyai et al. found that the amount of linalyl-acetate, one of the main components of clary sage, was 10.3% in the hydrodistilled oil, while in the SFE extract, it was only 8.2% [42]. The opposite trend was reported by Simándi et al., the amount of linalyl-acetate being 15.9% in the hydrodistilled oil, while in the SFE extract, it increased up to 23.6% [20]. The reason for the discrepancy is that different setting parameters were used during the SC-CO_2_ extraction. In order to control and compare our extraction processes, the effects of the setup parameters on the final product have to be clarified. A large number of parameters (pressure, temperature, cosolvent ratio, particle size, flow rate, extraction time, vessel shape, etc.) and a wide range of setting options would result in too many experiments. Using RSM in the present study allowed modeling the effects of three critical parameters against the studied respiratory pathogens.

The shape of the response surfaces is very similar in the two cases studied. This is a great advantage in optimization, because the ideal setting parameters for antibacterial activity are very close to each other for the two bacteria. It can be seen from our results that increasing the pressure has a beneficial effect on the antibacterial activity. The threshold where the biologically active components of the clary sage extract become soluble can be found at around 10 MPa. The higher pressure range is also more favorable from the aspect of the extraction yield [29,43], because with the increase in the pressure, the density of the liquid increases as well, which results an increased solubility of the components. Therefore, the higher the extraction pressure, the smaller the volume of fluid necessary for a given extraction [44]. However, it should be kept in mind that higher densities could increase the co-extraction of undesirable substances, which could alter the solubility of biologically active components. In our case, this did not occur at a pressure of 20 MPa yet.

However, a complete picture of the effect of pressure can only be obtained by examining it as the function of temperature. For both test bacteria, the modeling shows that a low temperature around 40 °C is preferred for antibacterial activity. This is probably due to the fact that the density of the fluid decreases with increasing temperature at constant pressure. As the compressibility of the fluid increases, the process becomes more pronounced [45]. The lower temperature is also much more advantageous in terms of avoiding thermodegradation [46], which is unavoidable when using hydrodistillation.

A cosolvent was used due to the extreme apolarity of SC-CO_2_ extract. Without the use of cosolvent, an increase in the ratio of vegetable fats and waxes was found in the preliminary experiments. This sometimes led to clogging of the back pressure regulator valve. The antibacterial activity of vegetable fats and waxes was relatively low, and therefore, their proportion was reduced by using a cosolvent. Ethanol was chosen due to its low toxicity and the fact that it dissolves the active components of EOs well even in the normal state. Although this parameter was the least important in terms of antibacterial activity (within the 1%–2% cosolvent ratio examined), it was on the other hand of great importance in the fine-tuning of our extraction. A polar modifier is necessary to improve the solubility of higher-weight and more polar molecules. In addition, the use of modifiers decreases the temperature required for the process [47].

Based on our results, RSM is suitable for understanding and optimizing supercritical extraction processes in terms of antibacterial activity (antibacterial effect), using appropriate analytical techniques. The modeling procedure that was developed can shorten the development of antibacterial products against *P. aeruginosa* and MRSA strains. In light of the results, clary sage can serve as an excellent raw material due to its exceptional phytochemical properties.

## 4. Materials and Methods

### 4.1. Plant Material—Salvia Sclarea

Plant material was ordered from Naturix24 Ltd. (Dransfeld, Germany) in 2019, who procure clary sage from Italy and the south of France within Europe (Naturix24, 2019). Their growing and harvesting of crops complies with the recommendations of Good Agricultural Practice. After harvest, the plant was air-dried, shredded, and shipped to Germany. The whole plant was processed. According to the statement of the merchant, the content of sclareol was relatively high, but the proportion of the compound in the essential oil was not more than 12%.

### 4.2. SFE Extraction

CO_2_ used for SFE was 99.97% (*w*/*w*) pure (Messer, Gödöllő, Hungary). The standard compound ethanol (Sigma-Aldrich, St. Louis, MO, USA) was used for the quantization. All solvents were of analytical grade and purchased from Sigma-Aldrich (St. Louis, MO, USA).

SFEs were performed on an SF2000 Able & Jasco instrument (Jasco, Tokyo, Japan). A 30 cm × 20 mm stainless steel column with a volume of 94.2 cm^3^ was used as the extraction vessel. Each vessel was loaded with 22 g of shredded, dried plants. The exact weight was recorded each time. As mobile phase, 99.9% pure CO_2_ was applied, and absolute ethanol was used as cosolvent. The proportion of ethanol varied between 1% and 2%. Each run lasted 120 min, as preliminary experiments showed that the extraction yield did not increase significantly with increasing the time further. The extracts were collected in 15 mL centrifuge tubes, the mass of which had already been weighed. Samples were stored at −10 °C until further processing.

In order to find the most effective method to reach the highest antibacterial effect, the extractions were performed by systematically changing the parameters according to the experiment plan. The parameters were determined following preliminary literature research [48,49,50,51,52]. The temperature ranged from 40 to 80 °C, the pressure ranged from 10 to 20 MPa, and the cosolvent ratio ranged from 1 to 2%. The color of the SC-CO_2_ extracts was from pale yellow to dark brown. The ethanol-insoluble portions were markedly separated in the extracts. Their scents were reminiscent of the starting material.

### 4.3. Determination of Ethanol Content of Samples with Gas Chromatography (GC-FID)

The analyses were carried out with an Agilent 6890N GC-FID (Santa Clara, CA, USA) system equipped with a TR-WAX (Thermo Fisher Scientific, Waltham, MA, USA) capillary column (30 m × 250 µm × 1.0 µm). The GC oven temperature was programmed to increase from 60 (5 min isothermal) to 240 °C at 30 °C/min (5 min isothermal). High-purity hydrogen (5.0) was used as a carrier gas at 2.9 mL/min (29 cm/s) in constant pressure mode. Vials were crimped in order to minimize the loss of volatile species. Absolute ethanol (a.r., Molar Chemicals Kft., Halásztelek, Hungary) was used as a standard to identify the ethanol peak based on retention time. For FID quantification, external standard technique was used. Then, 100 mg absolute ethanol was diluted with dimethyl sulfoxide (a.r., Molar Chemicals Kft., Halásztelek, Hungary) to achieve a final concentration of 10 mg/mL as a stock solution. The calibration curve covered the range 0.5 to 2.0 mg/mL.

### 4.4. Thin Layer Chromatography-Direct Bioautography (TLC-DB)

#### 4.4.1. Direct Bioautography

The antibacterial effect of the clary sage extracts on *P. aeruginosa* (ATCC 27853) and methicillin-resistant *Staphylococcus aureus* (MRSA 4262) was screened in the laboratory of the Department of Medical Microbiology and Immunology (Medical School, University of Pécs, Hungary). For bioautographic assay, bacteria were grown in 100 mL Brain–Heart Infusion Broth (BHI) (Sigma Aldrich Ltd., Darmstadt, Germany) at 37 °C in a shaker incubator at a speed of 60 rpm for 12 h [53]. The bacterial suspension was diluted with fresh nutrient BHI to an OD600 of 0.4, which corresponds to approximately 4 × 10^7^ colony-forming units (cfu)/ mL [16].

#### 4.4.2. Thin Layer Chromatography without Separation

Chromatography was performed on 5 × 10 cm silica gel 60F_254_ aluminum sheet TLC plates (Merck, Darmstadt, Germany). Chloroform (a.r., Molar Chemicals Kft., Halásztelek, Hungary) was chosen as the solvent because it dissolved the extracts well and had a high tension. Due to different extraction parameters, each sample had a different ethanol content (Table S1). During sample preparation, each sample was corrected for its ethanol content. From final solutions (final solution’s clary sage extract concentration in each sample: 10 mg/mL), 3.0 μL were applied to the TLC plate with Finnpipette pipettes (Thermo Fisher Scientific, Darmstadt, Germany); solvent controls were chloroform and absolute ethanol (a.r., Molar Chemicals Kft., Halásztelek, Hungary), while the positive controls were vancomycin (Vancocin, ANI Pharmaceuticals, Baudette, MN, USA) against MRSA (stock: 50 mg/mL; 0.6 μL applied to the TLC) and gentamicin (Sandoz, Holzkirchen, Germany) against *P. aeruginosa* (stock: 80 mg/2 mL; 0.75 μL applied to the TLC plate). TLC separation was not performed, because the goal of this experiment was to examine the antibacterial activity of the extracts (not separated compounds).

#### 4.4.3. Post-Chromatographic Detection

After sample application, the TLC plates were treated with the suspension of *P. aeruginosa* or MRSA. Layers were dipped into a 100 mL of bacterial suspension to assure a homogenous distribution and adhesion of bacteria onto the surface of the layers. After immersion, the layers were transferred into a low-wall horizontal chamber (with dimensions of 20 × 14.5 × 5 cm) and incubated for 2 h at 37 °C. Thereafter, for the visualization of antibacterial spots, TLC plates were immersed into the aqueous solution of 3-(4,5-dimethylthiazol-2-yl)-2,5-diphenyltetrazolium bromide (MTT, 0.05 g/85 mL) (Sigma Aldrich Ltd., Darmstadt, Germany) for 5 s and then incubated at 37 °C for 24 h. On the TLC plate, metabolically active bacteria convert the tetrazolium salt, MTT, into formazan dye. White spots (as inhibition zones) against the bluish-violet background indicated the lack of dehydrogenase activity due to the antibacterial activity of the tested samples [16]. All tests were carried out 6 times in parallel. The inhibitory zones (expressed in mm) of extracts were measured with Motic Images Plus 2.0 (Motic Deutschland GmbH, Wetzlar, Germany) program [16].

### 4.5. Experimental Design

The software Design Expert (Version 10, Stat-Ease Inc., Minneapolis, MN, USA) was applied for experimental design, data analysis, and model building. The factors used in the study were selected based on preliminary literature [31,54,55,56,57]. Factors are parameters that greatly influence the process, and factor levels are values that can be taken up by factors. The extraction pressure was variable A, the extraction temperature was variable B, and the cosolvent ratio was variable C. All variables were examined at 3 levels, and the dependent variable was antibacterial activity. A total of 27 extractions were performed. The investigated factors and levels tested are reported in Table 4. A full quadratic equation or the diminished form of this equation was used for this model [58,59]:

$$y=\;\beta_{0}+\overset{k}{\underset{i=1}{\sum }}\beta_{i}x_{i}+\overset{k}{\underset{i=1}{\sum }}\beta_{ii}x_{i}^{2}+\overset{k-1}{\underset{i=1}{\sum }}\overset{k}{\underset{j=2}{\sum }}\beta_{ij}x_{i}x_{j},\;\left(i<j\right)+\;\varepsilon$$

where *y* is the investigated response (antibacterial activity), *β*_0_, *β_i_*, *β_ii_*, and *β_ij_* are the constant regression coefficients, linear, quadratic, and interaction terms, respectively; *x_i_* and *x_j_* are coded independent variables, and *ε* is an unknown constant error vector [60]. Analysis of variance (ANOVA) was used to determine the quality of the fitted model. Statistical analyses were performed at the 95% confidence interval level.

## 5. Conclusions

Product development for the treatment of pathogens requires the use of a reproducible analytical method. The standardized TLC-DB method presented in this study is suitable for reproducible, quantitative monitoring of antibacterial activity against *P. aeruginosa* and MRSA. The correct choice of the combination of setting parameters during SC-CO_2_ extraction is critical. The antibacterial activity of the clary sage extract can be sensitively varied depending on the extraction conditions. The pressure and temperature used during the extraction had the greatest effect on the quality of the final product. RSM allows the optimization of SC-CO_2_ extraction and a deeper understanding of the process on a statistical basis. Further experiments (solid-phase microextraction SPME-gas chromatography-mass spectrometry (SPME-GC-MS) analysis) are needed to pair biological efficiency with chemical composition and adequate extraction yield. The optimal conditions, in the range of analyzed extraction parameters were calculated to be at a pressure of 18.6 MPa, a temperature of 40 °C, and an EtOH ratio of 2%. The extract obtained from clary sage with these adjusted optimal parameters of SCF extraction is of good quality and rich in EO; therefore, it is recommended for cosmetic and food use as well. In summary, this work presents an example using clary sage extract in relation to human health and pharmaceutical industry, but the combination of analytical and statistical methods could be useful on any kind of herbs in a wide range of use from perfume manufacturing to the food industry.

## Figures and Tables

**Table 1:** The inhibitory zones of the clary sage SFE extracts after TLC-DB and the extraction parameters of SCF procedure.

	*P. aeruginosa*		MRSA				
Sample	Diameter (mm)	^1^ SD	Diameter (mm)	^1^ SD	MPa	°C	EtOH (%)
**1**	5.86	0.72	5.46	0.50	20	40	2
**2**	5.50	0.56	4.85	0.78	15	40	2
**3**	7.51	0.85	7.57	0.62	10	40	2
**4**	6.18	0.64	4.69	0.46	20	60	2
**5**	5.43	0.42	4.80	0.38	15	60	2
**6**	0.00	0.00	0.00	0.00	10	60	2
**7**	4.29	0.71	4.66	0.39	20	80	2
**8**	4.83	0.29	4.32	0.59	15	80	2
**9**	0.00	0.00	0.00	0.00	10	80	2
**10**	6.50	0.42	4.59	0.65	20	40	1.5
**11**	6.91	0.20	4.77	0.69	15	40	1.5
**12**	6.27	0.33	3.29	0.42	10	40	1.5
**13**	5.41	0.47	3.41	0.36	20	60	1.5
**14**	5.71	0.80	4.25	0.35	15	60	1.5
**15**	0.00	0.00	0.00	0.00	10	60	1.5
**16**	6.37	0.78	3.50	0.40	20	80	1.5
**17**	6.56	0.55	3.29	0.46	15	80	1.5
**18**	0.00	0.00	0.00	0.00	10	80	1.5
**19**	5.96	0.15	4.44	0.53	20	40	1
**20**	6.34	0.82	3.58	0.82	15	40	1
**21**	5.32	0.41	3.72	0.40	10	40	1
**22**	5.61	0.47	3.72	0.40	20	60	1
**23**	4.98	0.61	3.07	0.36	15	60	1
**24**	0.00	0.00	0.00	0.00	10	60	1
**25**	3.02	0.23	3.40	0.58	20	80	1
**26**	2.75	0.48	3.25	0.68	15	80	1
**27**	0.00	0.00	0.00	0.00	10	80	1
**chloroform**	0.00	0.00	0.00	0.00			
**ch + EtOH**	0.00	0.00	0.00	0.00			
**EtOH**	0.00	0.00	0.00	0.00			
**Vancomycin**	-	-	12.30	0.45			
**Gentamicin**	8.26	0.81	-	-			

^1^ SD = standard deviation; ch = chloroform.

**Table 2:** Analysis of variance (ANOVA) for *P. aeruginosa* with quadratic model.

Source	Sum of Squares	df	Mean Square	F-Value	*p*-Value
**Model**	25.22	9	2.80	16.40	<0.0001
**A-Pressure**	9.94	1	9.94	58.15	<0.0001
**B-Temp.**	5.67	1	5.67	33.17	<0.0001
**C-EtOH**	0.1650	1	0.1650	0.9656	0.3365
**AB**	3.49	1	3.49	20.44	0.0002
**AC**	1.640 × 10^−6^	1	1.640 × 10^−6^	9.597 × 10^−6^	0.9976
**BC**	0.0046	1	0.0046	0.0269	0.8712
**A^2^**	4.90	1	4.90	28.66	<0.0001
**B^2^**	0.9233	1	0.9233	5.40	0.0297
**C^2^**	0.2831	1	0.2831	1.66	0.2114

R^2^ = 0.8703, Adjusted R^2^ = 0.8172, Predicted R^2^ = 0.662, Significant ≤ 0.050.

**Table 3:** Analysis of variance (ANOVA) for MRSA with quadratic model.

Source	Sum of Squares	df	Mean Square	F-Value	*p*-Value
**Model**	19.70	9	2.19	17.91	<0.0001
**A-Pressure**	7.88	1	7.88	64.47	<0.0001
**B-Temp.**	3.86	1	3.86	31.63	0.0001
**C-EtOH**	0.5615	1	0.5615	4.59	0.0434
**AB**	2.78	1	2.78	22.78	<0.0001
**AC**	0.0001	1	0.0001	0.0012	0.9724
**BC**	0.1450	1	0.1450	1.19	0.2878
**A^2^**	4.18	1	4.18	34.18	<0.0001
**B^2^**	0.7909	1	0.7909	6.47	0.0185
**C^2^**	0.0086	1	0.0086	0.0708	0.7927

R^2^ = 0.8799, Adjusted R^2^ = 0.8308, Predicted R^2^ = 0.6860, Significant ≤ 0.050.

**Table 4:** Coded and real levels of independent variables for the designed experiment.

Independent Variable	Symbol	Levels		
		Low (−1)	Middle (0)	High (+1)
Pressure (MPa)	A	10	15	20
Temperature (°C)	B	40	60	80
Cosolvent (%)	C	1.0	1.5	2.0

## Data Availability

Data supporting the reported results will be available from the authors.

## Supplementary Materials

The following supplementary material is available online. Table S1: The ethanol content and extraction yield of the clary sage SFE extracts.

## Sample Availability

Not available.
