# Supplementary material for: Combination of Analytical and Statistical Methods in Order to Optimize Antibacterial Activity of Clary Sage Supercritical Fluid Extracts
Source: Molecules. 2021 Oct 26;26(21):6449. doi: 10.3390/molecules26216449 (PMC8586929; doi:10.3390/molecules26216449)
Supplement: Supplementary file 1 [file molecules-26-06449-s001.zip › molecules-1414810-supplementary.pdf]

Supplementary information

Table 1. The ethanol content and extraction yield of the clary sage SFE extracts.

Sample	Extract + EtOH (g)	EtOH (m/m%)	Extraction yield (g)	Extraction yield (m/m%)
**1**	6.794	83.31	1.13	5.12
**2**	5.1064	88.72	0.58	2.68
**3**	3.9711	94.75	0.21	0.97
**4**	5.0402	84.48	0.78	3.70
**5**	5.757	85.15	0.85	3.96
**6**	4.8512	88.19	0.57	2.84
**7**	6.6173	86.42	0.90	4.45
**8**	4.116	91.78	0.34	1.70
**9**	6.2144	93.92	0.38	1.90
**10**	4.9519	85.05	0.74	3.51
**11**	6.6124	89.55	0.69	3.33
**12**	4.8933	87.10	0.63	2.94
**13**	4.0805	79.60	0.83	4.08
**14**	5.3441	87.87	0.65	3.05
**15**	1.9945	86.31	0.27	1.36
**16**	3.8984	79.71	0.79	3.75
**17**	3.835	85.67	0.55	2.61
**18**	3.3985	85.48	0.49	2.17
**19**	6.7074	76.87	1.55	7.37
**20**	5.3505	75.38	1.32	6.40
**21**	4.1491	76.68	0.97	4.73
**22**	3.8839	81.51	0.72	3.48
**23**	3.8192	78.77	0.81	3.89
**24**	3.4193	88.26	0.40	1.92
**25**	2.9835	75.13	0.74	3.57
**26**	4.3975	77.56	0.99	4.50
**27**	3.3701	76.50	0.79	3.54
